# Supplementary material for: Fluorescence Microscopy in Adeno-Associated Virus Research
Source: Viruses. 2023 May 16;15(5):1174. doi: 10.3390/v15051174 (PMC10222864; doi:10.3390/v15051174)
Supplement: Supplementary file 1 [file viruses-15-01174-s001.zip › viruses-2313188-supplementary.pdf]

viruses

Review

Fluorescence Microscopy in Adeno-Associated Virus Research

Susanne K. Golm, Wolfgang Hübner and Kristian M. Müller

Supplementary Information

Table S1. Examples of microscopy techniques in the context of AAV visualization with fluorophores.

Labeling	Micros-cope	Details	Con-text	Cells/Tissue	Topic	Authors	Year	Ref.
aa-reactive dyes	Widefield	Cy3, AAV2, Ad	Td[a]	male rats, inferior col- liculus or hypocampus	Tropism of AAV2	Bartlett et al.	1998	[1]
aa-reactive dyes	Widefield Confocal	Cy2-AAV3, Cy3-AAV3, Cy3-AAV2, Cy2-AAV3, Cy3-Ad	Td	HeLa	Colocalization of AAV2 and AAV3, not of AAV and Ad	Bartlett et al.	2000	[2]
aa-reactive dyes	Confocal	Cy5-labeld AAV	Td	HeLa	Single virus tracing, from endoctosis to nucleus	Seisenberger et al.	2001	[3]
aa-reactive dyes	Confocal	AlexaAluor488-AAV2	Td	HeLa	Influence of Ad coinfection	Xiao et al.	2002	[4]
aa-reactive dyes	Confocal	Cy3-AAV1,2,5,8 and 9	Td	Retina of rats	Improvement of AAV infection of different serotypes by co application of Pronase	Dalkara et al.	2009	[5]
aa-reactive dyes	Confocal	AlexaFluor 488-AAV2 (QD AAV2)	Td	HeLa	Colocalization studies, trafficking, new technique (QD)	Joo et al.	2011	[6]
aa-reactive dye, Antibody	Confocal AFM	Cy5-AAV2, A20	Td[a]	HeLa, mouse muscle	Deconvolution, AAV trafficking, AAV count	Xiao et al.	2012	[7]
aa-reactive dyes	Confocal	Cy5-AAV2	Td	HeLa	AAV nuclear entry	Nicolson et al.	2014	[8]
aa-reactive dyes	Confocal	AAV2-Luc-AlexaFluor 568	Td	HeLa	AAV binding to AAVR	Cui et al.	2019	[9]
aa-reactive dyes	Confocal	Cy5-AAV2	Td	HeLa	Improvement of transduction, capsid modification without genetical modification	Mével et al.	2020	[10]
Antibody	Widefield	Anti-Rep:78/68 Anti-Rep: 52/40	Td	D6	Colocalization of Rep and Cap in the nucleous	Hunter et al.	1992	[11]
Antibody	Confocal	A1, A69, B1, A20, AAV2	Td	HeLa	Rep and Cap tracing	Wistuba et al.	1997	[12]
Antibody	Widefield	A20, AAV2	Td	HEK293	Colocalization of AAV2 and nucleolin	Qiu et al.	1999	[13]

Labeling	Micros-cope	Details	Con-text	Cells/Tissue	Topic	Authors	Year	Ref.
Antibody	Widefield	C24-B, C37-B, A20, D3	Td	HeLa	Epitope mapping and cell interaction	Worbus et al.	2000	[14]
Antibody	Widefield	Anti-Rep	Td	BHK	ssDNA-dependent colocalization of AAV Rep and HSV ICP8 in nuclear replication domains	Heilbronn et al.	2003	[15]
Antibody, DNA-labeling	Widefield	Anti-Rep, BrU-labeld DNA+BrU-Antibody	Td/Tf	HeLa	Rep interactions, colocalization studies	Stracker et al.	2004	[16]
Antibody	Confocal	ADK6, ADK8, ADK9; AAV1,4,5 or 6	Tf	911	AAV serotype specific ELISAs	Kuck et al.	2006	[17]
Antibody	Confocal	Anti-Rep	Tf	HeLa	Role of HSV Helicase-Primase complex during AAV DNA replication	Slanina et al.	2006	[18]
Antibody, aa-reactive dyes	Confocal	A20, AlexaFluor	Td	HeLa	NPM plays a role in AAV amplification affecting Rep	Bevington	2007	[19]
Antibody	Confocal	A20	Td	HeLa	Identification of cellular parameters that mobilize virions in the nucleus and significantly influence AAV infection	Johnson et al.	2009	[20]
Antibody	Confocal	A20, A1/αVP1, B1	Td	HeLa	localize full and empty AAV capsids relative to nucleolin with confocal microscopy and z-stacks as well as the effect of hydroxyurea or the proteasome inhibitor MG132 on AAV-2 localization	Johnson et al.	2010	[21]
Antibody	Confocal	ADK6, ADK8, ADK9	Tf[a]	HeLa	Role of AAP for different serotypes	Sonntag et al.	2011	[22]
Antibody	Cryo-EM	A20,	-	-	Structure of AAV2-A20	McCraw et al.	2012	[23]
Antibody	Cryo-EM, Confocal	ADK8	Td	HeLa	Epitope mapping on AAV8	Gurda et al.	2012	[24]
Antibody	Cryo-EM	4E4, 5H7, C37-B, 3C5	-	-	Epitope characterization	Gurda et al.	2013	[25]
Antibody	Cryo-EM	ADK1a, ADK1b, ADK5a, ADK5b	Td	293T, COS	AAV-monoclonal antibodyfragment complexes	Tseng et al.	2015	[26]
Antibody	Confocal	A20	Td	HeLa	Chemical modulation of endocytotic sorting of AAV	Berry et al.	2016	[27]

Labeling	Micros-cope	Details	Con-text	Cells/Tissue	Topic	Authors	Year	Ref.
Antibody, Tag-labeling	Confocal	A20, ADK4, ADK5a, ADK8, ADK8/9, AAP-FLAG	Tf	HeLa	VP3 of AVV 4, 5, and 11 assembled without requiring AAP	Earley et al.	2017	[28]
Antibody	Cryo-EM	ADK6	Td	HEK293T	Role of AAV6 K531 in receptor and ADK6 recognition	Bennett et al.	2018	[29]
Antibody	Cryo-EM	PAV9.1	Td	HEK293	Epitope mapping on AAV9	Giles et al.	2018	[30]
Antibody	Confocal	A20	Td	Human monocyte-derived immature DC (iDC)	Improvement of iDC transduction	Rossi et al.	2019	[31]
Antibody	Confocal	A20	Td	Huh7	(RNF121) a protein identified as regulator of AAV transcription	Madigan et al.	2019	[32]
Antibody	Cryo-EM	HL2476	Td	HEK293	Epitope characterization	Jose et al.	2019	[33]
Antibody	Widefield	A20, scFv A20	Td	HeLa	VLP production in *E.coli* and chemically defined capsid assembly	Le et al.	2019	[34]
Antibody	Confocal	A20	Td	Scr and ATP2C1 KO	altered trafficking	Madigan et al.	2020	[35]
Antibody	Confocal	Anti-MAAP GAL-KKI, B1	Tf	293 T	Functional roles of MAAP	Galibert et al.	2021	[36]
Antibody	Widefield	ADK5a	Td	HeLa	VLP production in *E.coli*	Le et al.	2022	[37]
Click chemistry	Confocal	DBIO-tagged fluorophore+ modified AAV2	Td	HeLa	Colocalization studies, early, late and recycling endosomes involved in AAV transduction	Zhang et al.	2018	[38]
Click chemistry	Confocal	DBIO-tagged fluorophore+ modified AAV-DJ	Td	HEK-293T	Improving AAV transduction, viral shielding via lipid based cloaks	Kartekar et al.	2018	[39]
DNA-staining, Antibody	Confocal	In situ hybridization, Anti-Rep	Td	HeLa	AAV depends on Ad coinfection	Weitzman et al.	1996	[40]
DNA-staining Antibody	Confocal	LacI-eYFP/lacO system, Anti-Rep	Td	HeLa	AAV is targeted by MRN and has evolved to exploit adenoviral proteins that degrade these cellular factors	Schwartz et al.	2007	[41]
DNA-staining	Confocal	LacI-eYFP/lacO system	Td	HeLa	Colocaization of DNA-damage-response proteins and rAAV genome processing	Cervelli et al.	2008	[42]
DNA and RNA-staining	Bight field	RNAscope	Td	Different mice tissues	DNA Distribution in Cellular Compartments and	Zhao et al.	2020	[43]

Labeling	Micros-cope	Details	Con-text	Cells/Tissue	Topic	Authors	Year	Ref.
					Tissues following Local and Systemic Injection , bilateral TA injections			
DNA-staining	Confocal	SABER-FISH	Td	microglia in the retina of CX3CR1$^{GFP/+}$ mice	In Situ Detection of Adeno-associated Viral Vector Genomes	Wang et al.	2020	[44]
DNA-staining, Antibody	Confocal	in situ hybridization, A20, A96, VP51	Td	NHF	uncoating of AAV2 particles occurs in a stepwise process that is completed in the nucleolus and supported by alteration of the nucleolar structure	Sutter	2022	[45]
Fusionprotein, Antibody	Widefield	Rep-GFP, Anti-Rep	Tf	HeLa	Characterization of NLS in c-terminus of Rep68/78	Cassell et al.	2004	[46]
Fusionprotein, DNA-labeling	Confocal	Rep-DsRed2, LacI-eYFP/lacO system	Tf	VERO cells, HeLa	AAV DNA replication in live cells	Fraefel et al.	2004	[47]
Fusionprotein, Antibody	Confocal	VP2-GFP, A20	Tf/Td	HeLa	Viral trafficking	Lux et al.	2005	[48]
Fusionprotein, Antibody, DNA-labeling	Confocal	Rep-eCFP, Anti-Rep, LacI-eYFP/lacO system	Tf	HeLa	independent replication from p5 and ITR	Glauser et al.	2005	[49]
Fusionprotein	Confocal	Mosaic AAV2-mCherry	Td	HeLa	Infective AAV2-VP3-mCherry	Judd et al.	2012	[50]
Fusionprotein, Tag-labeling	Confocal	GFP-AAP2, FLAG-AAP2	Tf	HeLa	Nuclear and nucleolar localization signals in AAV2 AAP	Earley et al.	2015	[51]
Tag-labeled	Widefield	biotin acceptor peptide (BAP) in AAV1 capsid	Td	*In vitro*: HeLa C12, human umbilical vein endothelial cells	Ligand conjugation for improved Transduction	Stachler et al.	2008	[52]
Tag-labeled	SPEED	AAV-biotin	Td	HeLa	Nuclear import, live cells	Kelich et al.	2015	[53]
Tag-labeled	Confocal	Tetracysteine-AAV9 capsid + fluorescent dye or biotin, maleimide-546 dye, maleimide-660-C2 dye	Td	*In vitro*: HEK293T *In vivo*: mice, 12 weeks old, facial vein, CNS	Capsid interactome	Chandran et al.	2017	[54]

Labeling	Micros-cope	Details	Con-text	Cells/Tissue	Topic	Authors	Year	Ref.
Tag-labeled	SPEED	AAV-BirA, AAV-AVI	Td	HeLa	Nuclear import, approximately 17% of AAV2 capsids can be intact and successfully imported through the NPC to reach the nucleus	Junod et al	2021	[55]
Tag-labeled, Fusionprotein	Confocal	MAAP-eGFP Anti-MAAP8-HA-A647	Tf	HEK293	Serotype dependent AAV secretion	Elmore et al.	2021	[56]

[a] Td: Transduction, TF: Transfection

References

(note: for clarity, references are numbered here according to the supplementary text)

1. Bartlett, J.S.; Samulski, R.J.; McCown, T.J. Selective and Rapid Uptake of Adeno-Associated Virus Type 2 in Brain. *Hum. Gene Ther.* **1998**, *9*, 1181–1186, doi:10.1089/hum.1998.9.8-1181.
2. Bartlett, J.S.; Wilcher, R.; Samulski, R.J. Infectious Entry Pathway of Adeno-Associated Virus and Adeno-Associated Virus Vectors. *J. Virol.* **2000**, *74*, 2777–2785, doi:10.1128/jvi.74.6.2777-2785.2000.
3. Seisenberger, G.; Ried, M.U.; Endreß, T.; Büning, H.; Hallek, M.; Bräuchle, C. Real-Time Single-Molecule Imaging of the Infection Pathway of an Adeno-Associated Virus. *Science* **2001**, *294*, 1929–1932, doi:10.1126/science.1064103.
4. Xiao, W.; Warrington, K.H.; Hearing, P.; Hughes, J.; Muzyczka, N. Adenovirus-Facilitated Nuclear Translocation of Adeno-Associated Virus Type 2. *J. Virol.* **2002**, *76*, 11505–11517, doi:10.1128/JVI.76.22.11505-11517.2002.
5. Dalkara, D.; Kolstad, K.D.; Caporale, N.; Visel, M.; Klimczak, R.R.; Schaffer, D. V.; Flannery, J.G. Inner Limiting Membrane Barriers to AAV-Mediated Retinal Transduction From the Vitreous. *Mol. Ther.* **2009**, *17*, 2096–2102, doi:10.1038/mt.2009.181.
6. Joo, K.; Fang, Y.; Liu, Y.; Xiao, L.; Gu, Z.; Tai, A.; Lee, C.; Tang, Y.; Wang, P. Enhanced Real-Time Monitoring of Adeno-Associated Virus Trafficking by Virus-Quantum Dot Conjugates. *ACS Nano* **2011**, *5*, 3523–3535, doi:10.1021/nn102651p.
7. Xiao, P.-J.; Li, C.; Neumann, A.; Samulski, R.J. Quantitative 3D Tracing of Gene-Delivery Viral Vectors in Human Cells and Animal Tissues. *Mol. Ther.* **2012**, *20*, 317–328, doi:10.1038/mt.2011.250.
8. Nicolson, S.C.; Samulski, R.J. Recombinant Adeno-Associated Virus Utilizes Host Cell Nuclear Import Machinery To Enter the Nucleus. *J. Virol.* **2014**, *88*, 4132–4144, doi:10.1128/jvi.02660-13.
9. Cui, M.; Lu, Y.; Tang, C.; Zhang, R.; Wang, J.; Si, Y.; Cheng, S.; Ding, W. A Generic Method for Fast and Sensitive Detection of Adeno-Associated Viruses Using Modified AAV Receptor Recombinant Proteins. *Molecules* **2019**, *24*, 3973, doi:10.3390/molecules24213973.
10. Mével, M.; Bouzelha, M.; Leray, A.; Pacouret, S.; Guilbaud, M.; Penaud-Budloo, M.; Alvarez-Dorta, D.; Dubreil, L.; Gouin, S.G.; Combal, J.P.; et al. Chemical Modification of the Adeno-Associated Virus Capsid to Improve Gene Delivery. *Chem. Sci.* **2020**, *11*, 1122–1131, doi:10.1039/c9sc04189c.
11. Hunter, L.A.; Samulski, R.J. Colocalization of Adeno-Associated Virus Rep and Capsid Proteins in the Nuclei of Infected Cells. *J. Virol.* **1992**, *66*, 317–324.
12. Wistuba, A.; Kern, A.; Weger, S.; Grimm, D.; Kleinschmidt, J.A. Subcellular Compartmentalization of Adeno-Associated Virus Type 2 Assembly. *J. Virol.* **1997**, *71*, 1341–1352, doi:10.1128/jvi.71.2.1341-1352.1997.

13. Qiu, J.; Brown, K.E. A 110-KDa Nuclear Shuttle Protein, Nucleolin, Specifically Binds to Adeno-Associated Virus Type 2 (AAV-2) Capsid. *Virology* **1999**, *257*, 373–382.
14. Wobus, C.E.; Hügle-Dörr, B.; Girod, A.; Petersen, G.; Hallek, M.; Kleinschmidt, J.A. Monoclonal Antibodies against the Adeno-Associated Virus Type 2 (AAV-2) Capsid: Epitope Mapping and Identification of Capsid Domains Involved in AAV-2–Cell Interaction and Neutralization of AAV-2 Infection. *J. Virol.* **2000**, *74*, 9281–9293, doi:10.1128/JVI.74.19.9281-9293.2000.
15. Heilbronn, R.; Engstler, M.; Weger, S.; Krahn, A.; Schetter, C.; Boshart, M. SsDNA-Dependent Colocalization of Adeno-Associated Virus Rep and Herpes Simplex Virus ICP8 in Nuclear Replication Domains. *Nucleic Acids Res.* **2003**, *31*, 6206–6213, doi:10.1093/nar/gkg827.
16. Stracker, T.H.; Cassell, G.D.; Ward, P.; Loo, Y.-M.; van Breukelen, B.; Carrington-Lawrence, S.D.; Hamatake, R.K.; van der Vliet, P.C.; Weller, S.K.; Melendy, T.; et al. The Rep Protein of Adeno-Associated Virus Type 2 Interacts with Single-Stranded DNA-Binding Proteins That Enhance Viral Replication. *J. Virol.* **2004**, *78*, 441–453, doi:10.1128/jvi.78.1.441-453.2004.
17. Kuck, D.; Kern, A.; Kleinschmidt, J.A. Development of AAV Serotype-Specific ELISAs Using Novel Monoclonal Antibodies. *J. Virol. Methods* **2006**, *140*, 17–24, doi:10.1016/j.jviromet.2006.10.005.
18. Slanina, H.; Weger, S.; Stow, N.D.; Kuhrs, A.; Heilbronn, R. Role of the Herpes Simplex Virus Helicase-Primase Complex during Adeno-Associated Virus DNA Replication. *J. Virol.* **2006**, *80*, 5241–5250, doi:10.1128/JVI.02718-05.
19. Bevington, J.M.; Needham, P.G.; Verrill, K.C.; Collaco, R.F.; Basrur, V.; Trempe, J.P. Adeno-Associated Virus Interactions with B23/Nucleophosmin: Identification of Sub-Nucleolar Virion Regions. *Virology* **2007**, *357*, 102–113, doi:10.1016/j.virol.2006.07.050.
20. Johnson, J.S.; Samulski, R.J. Enhancement of Adeno-Associated Virus Infection by Mobilizing Capsids into and out of the Nucleolus. *J. Virol.* **2009**, *83*, 2632–2644, doi:10.1128/JVI.02309-08.
21. Johnson, J.S.; Li, C.; DiPrimio, N.; Weinberg, M.S.; McCown, T.J.; Samulski, R.J. Mutagenesis of Adeno-Associated Virus Type 2 Capsid Protein VP1 Uncovers New Roles for Basic Amino Acids in Trafficking and Cell-Specific Transduction. *J. Virol.* **2010**, *84*, 8888–8902, doi:10.1128/JVI.00687-10.
22. Sonntag, F.; Köther, K.; Schmidt, K.; Weghofer, M.; Raupp, C.; Nieto, K.; Kuck, A.; Gerlach, B.; Böttcher, B.; Müller, O.J.; et al. The Assembly-Activating Protein Promotes Capsid Assembly of Different Adeno-Associated Virus Serotypes. *J. Virol.* **2011**, *85*, 12686–12697, doi:10.1128/JVI.05359-11.
23. McCraw, D.M.; O'Donnell, J.K.; Taylor, K.A.; Stagg, S.M.; Chapman, M.S. Structure of Adeno-Associated Virus-2 in Complex with Neutralizing Monoclonal Antibody A20. *Virology* **2012**, *431*, 40–49, doi:10.1016/j.virol.2012.05.004.
24. Gurda, B.L.; Raupp, C.; Popa-Wagner, R.; Naumer, M.; Olson, N.H.; Ng, R.; McKenna, R.; Baker, T.S.; Kleinschmidt, J.A.; Agbandje-McKenna, M. Mapping a Neutralizing Epitope onto the Capsid of Adeno-Associated Virus Serotype 8. *J. Virol.* **2012**, *86*, 7739–7751, doi:10.1128/jvi.00218-12.
25. Gurda, B.L.; DiMattia, M.A.; Miller, E.B.; Bennett, A.; McKenna, R.; Weichert, W.S.; Nelson, C.D.; Chen, W.; Muzyczka, N.; Olson, N.H.; et al. Capsid Antibodies to Different Adeno-Associated Virus Serotypes Bind Common Regions. *J. Virol.* **2013**, *87*, 9111–9124, doi:10.1128/jvi.00622-13.
26. Tseng, Y.-S.; Gurda, B.L.; Chipman, P.; McKenna, R.; Afione, S.; Chiorini, J.A.; Muzyczka, N.; Olson, N.H.; Baker, T.S.; Kleinschmidt, J.; et al. Adeno-Associated Virus Serotype 1 (AAV1)- and AAV5-Antibody Complex Structures Reveal Evolutionary Commonalities in Parvovirus Antigenic Reactivity. *J. Virol.* **2015**, *89*, 1794–1808, doi:10.1128/jvi.02710-14.
27. Berry, G.E.; Asokan, A. Chemical Modulation of Endocytic Sorting Augments Adeno-Associated Viral Transduction. *J. Biol. Chem.* **2016**, *291*, 939–947, doi:10.1074/jbc.M115.687657.
28. Earley, L.F.; Powers, J.M.; Adachi, K.; Baumgart, J.T.; Meyer, N.L.; Xie, Q.; Chapman, M.S.; Nakai, H. Adeno-Associated Virus (AAV) Assembly-Activating Protein Is Not an Essential Requirement for Capsid Assembly of AAV Serotypes 4, 5, and 11. *J. Virol.* **2017**, *91*, e01980-16, doi:10.1128/JVI.01980-16.

29. Bennett, A.D.; Wong, K.; Lewis, J.; Tseng, Y.; Smith, J.K.; Chipman, P.; McKenna, R.; Samulski, R.J.; Kleinschmidt, J.; Agbandje-McKenna, M. AAV6 K531 Serves a Dual Function in Selective Receptor and Antibody ADK6 Recognition. *Virology* **2018**, *518*, 369–376, doi:10.1016/j.virol.2018.03.007.
30. Giles, A.R.; Govindasamy, L.; Somanathan, S.; Wilson, J.M. Mapping an Adeno-Associated Virus 9-Specific Neutralizing Epitope To Develop Next-Generation Gene Delivery Vectors. *J. Virol.* **2018**, *92*, e010011-18, doi:10.1128/JVI.01011-18.
31. Rossi, A.; Dupaty, L.; Aillot, L.; Zhang, L.; Gallien, C.; Hallek, M.; Odenthal, M.; Adriouch, S.; Salvetti, A.; Büning, H. Vector Uncoating Limits Adeno-Associated Viral Vector-Mediated Transduction of Human Dendritic Cells and Vector Immunogenicity. *Sci. Rep.* **2019**, *9*, 3631, doi:10.1038/s41598-019-40071-1.
32. Madigan, V.J.; Yuziuk, J.A.; Chiarella, A.M.; Tyson, T.O.; Meganck, R.M.; Elmore, Z.C.; Tse, L. V.; Hathaway, N.A.; Asokan, A. Ring Finger Protein 121 Is a Potent Regulator of Adeno-Associated Viral Genome Transcription. *PLOS Pathog.* **2019**, *15*, e1007988, doi:10.1371/journal.ppat.1007988.
33. Jose, A.; Mietzsch, M.; Smith, J.K.; Kurian, J.; Chipman, P.; McKenna, R.; Chiorini, J.; Agbandje-McKenna, M. High-Resolution Structural Characterization of a New Adeno-Associated Virus Serotype 5 Antibody Epitope toward Engineering Antibody-Resistant Recombinant Gene Delivery Vectors. *J. Virol.* **2019**, *93*, e01394-18, doi:10.1128/JVI.01394-18.
34. Le, D.T.; Radukic, M.T.; Müller, K.M. Adeno-Associated Virus Capsid Protein Expression in Escherichia Coli and Chemically Defined Capsid Assembly. *Sci. Rep.* **2019**, *9*, 18631, doi:10.1038/s41598-019-54928-y.
35. Madigan, V.J.; Berry, G.E.; Tyson, T.O.; Nardone-White, D.; Ark, J.; Elmore, Z.C.; Murlidharan, G.; Vincent, H.A.; Asokan, A. The Golgi Calcium ATPase Pump Plays an Essential Role in Adeno-Associated Virus Trafficking and Transduction. *J. Virol.* **2020**, *94*, e01604-20, doi:10.1128/JVI.01604-20.
36. Galibert, L.; Hyvönen, A.; Eriksson, R.A.E.; Mattola, S.; Aho, V.; Salminen, S.; Albers, J.D.; Peltola, S.K.; Weman, S.; Nieminen, T.; et al. Functional Roles of the Membrane-Associated AAV Protein MAAP. *Sci. Rep.* **2021**, *11*, 21698, doi:10.1038/s41598-021-01220-7.
37. Le, D.T.; Radukic, M.T.; Teschner, K.; Becker, L.; Müller, K.M. Synthesis and Concomitant Assembly of Adeno-Associated Virus-like Particles in Escherichia Coli. *ACS Synth. Biol.* **2022**, *11*, 3601–3607, doi:10.1021/acssynbio.2c00451.
38. Zhang, C.; Zhou, X.; Yao, T.; Tian, Z.; Zhou, D. Precision Fluorescent Labeling of an Adeno-Associated Virus Vector to Monitor the Viral Infection Pathway. *Biotechnol. J.* **2018**, *13*, e1700374, doi:10.1002/biot.201700374.
39. Katrekar, D.; Moreno, A.M.; Chen, G.; Worlikar, A.; Mali, P. Oligonucleotide Conjugated Multi-Functional Adeno-Associated Viruses. *Sci. Rep.* **2018**, *8*, 3589, doi:10.1038/s41598-018-21742-x.
40. Weitzman, M.D.; Fisher, K.J.; Wilson, J.M. Recruitment of Wild-Type and Recombinant Adeno-Associated Virus into Adenovirus Replication Centers. *J. Virol.* **1996**, *70*, 1845–1854, doi:10.1128/jvi.70.3.1845-1854.1996.
41. Schwartz, R.A.; Palacios, J.A.; Cassell, G.D.; Adam, S.; Giacca, M.; Weitzman, M.D. The Mre11/Rad50/Nbs1 Complex Limits Adeno-Associated Virus Transduction and Replication. *J. Virol.* **2007**, *81*, 12936–12945, doi:10.1128/JVI.01523-07.
42. Cervelli, T.; Palacios, J.A.; Zentilin, L.; Mano, M.; Schwartz, R.A.; Weitzman, M.D.; Giacca, M. Processing of Recombinant AAV Genomes Occurs in Specific Nuclear Structures That Overlap with Foci of DNA-Damage-Response Proteins. *J. Cell Sci.* **2008**, *121*, 349–357, doi:10.1242/jcs.003632.
43. Zhao, J.; Yue, Y.; Patel, A.; Wasala, L.; Karp, J.F.; Zhang, K.; Duan, D.; Lai, Y. High-Resolution Histological Landscape of AAV DNA Distribution in Cellular Compartments and Tissues Following Local and Systemic Injection. *Mol. Ther. - Methods Clin. Dev.* **2020**, *18*, 856–868, doi:10.1016/j.omtm.2020.08.006.
44. Wang, S.K.; Lapan, S.W.; Hong, C.M.; Krause, T.B.; Cepko, C.L. In Situ Detection of Adeno-Associated Viral Vector Genomes with SABER-FISH. *Mol. Ther. - Methods Clin. Dev.* **2020**, *19*, 376–386, doi:10.1016/j.omtm.2020.10.003.

45. Sutter, S.O.; Lkharrazi, A.; Schraner, E.M.; Michaelsen, K.; Meier, A.F.; Marx, J.; Vogt, B.; Büning, H.; Fraefel, C. Adeno-Associated Virus Type 2 (AAV2) Uncoating Is a Stepwise Process and Is Linked to Structural Reorganization of the Nucleolus. *PLOS Pathog.* **2022**, *18*, e1010187, doi:10.1371/journal.ppat.1010187.
46. Cassell, G.D.; Weitzman, M.D. Characterization of a Nuclear Localization Signal in the C-Terminus of the Adeno-Associated Virus Rep68/78 Proteins. *Virology* **2004**, *327*, 206–214, doi:10.1016/j.virol.2004.06.034.
47. Fraefel, C.; Bittermann, A.G.; Büeler, H.; Heid, I.; Bächi, T.; Ackermann, M. Spatial and Temporal Organization of Adeno-Associated Virus DNA Replication in Live Cells. *J. Virol.* **2004**, *78*, 389–398, doi:10.1128/JVI.78.1.389.
48. Lux, K.; Goerlitz, N.; Schlemminger, S.; Perabo, L.; Goldnau, D.; Endell, J.; Leike, K.; Kofler, D.M.; Finke, S.; Hallek, M. Green Fluorescent Protein-Tagged Adeno-Associated Virus Particles Allow the Study of Cytosolic and Nuclear Trafficking. *J. Virol.* **2005**, *79*, 11776–11787, doi:10.1128/JVI.79.18.11776–11787.2005.
49. Glauser, D.L.; Saydam, O.; Balsiger, N.A.; Heid, I.; Linden, R.M.; Ackermann, M.; Fraefel, C. Four-Dimensional Visualization of the Simultaneous Activity of Alternative Adeno-Associated Virus Replication Origins. *J. Virol.* **2005**, *79*, 12218–12230, doi:10.1128/JVI.79.19.12218-12230.2005.
50. Judd, J.; Wei, F.; Nguyen, P.Q.; Tartaglia, L.J.; Agbandje-McKenna, M.; Silberg, J.J.; Suh, J. Random Insertion of MCherry Into VP3 Domain of Adeno-Associated Virus Yields Fluorescent Capsids With No Loss of Infectivity. *Mol. Ther. Nucleic Acids* **2012**, *1*, e54, doi:10.1038/mtna.2012.46.
51. Earley, L.F.; Kawano, Y.; Adachi, K.; Sun, X.-X.; Dai, M.-S.; Nakai, H. Identification and Characterization of Nuclear and Nucleolar Localization Signals in the Adeno-Associated Virus Serotype 2 Assembly-Activating Protein. *J. Virol.* **2015**, *89*, 3038–3048, doi:10.1128/jvi.03125-14.
52. Stachler, M.D.; Chen, I.; Ting, A.Y.; Bartlett, J.S. Site-Specific Modification of AAV Vector Particles with Biophysical Probes and Targeting Ligands Using Biotin Ligase. *Mol. Ther.* **2008**, *16*, 1467–1473, doi:10.1038/mt.2008.129.
53. Kelich, J.M.; Ma, J.; Dong, B.; Wang, Q.; Chin, M.; Magura, C.M.; Xiao, W.; Yang, W. Super-Resolution Imaging of Nuclear Import of Adeno-Associated Virus in Live Cells. *Mol. Ther. - Methods Clin. Dev.* **2015**, *2*, 15047, doi:10.1038/mtm.2015.47.
54. Chandran, J.S.; Sharp, P.S.; Karyka, E.; Aves-Cruzeiro, J.M.D.C.; Coldicott, I.; Castelli, L.; Hautbergue, G.; Collins, M.O.; Azzouz, M. Site Specific Modification of Adeno-Associated Virus Enables Both Fluorescent Imaging of Viral Particles and Characterization of the Capsid Interactome. *Sci. Rep.* **2017**, *7*, 14766, doi:10.1038/s41598-017-15255-2.
55. Junod, S.L.; Saredy, J.; Yang, W. Nuclear Import of Adeno-Associated Viruses Imaged by High-Speed Single-Molecule Microscopy. *Viruses* **2021**, *13*, 167, doi:10.3390/v13020167.
56. Elmore, Z.C.; Patrick Havlik, L.; Oh, D.K.; Anderson, L.; Daaboul, G.; Devlin, G.W.; Vincent, H.A.; Asokan, A. The Membrane Associated Accessory Protein Is an Adeno-Associated Viral Egress Factor. *Nat. Commun.* **2021**, *12*, 1–13, doi:10.1038/s41467-021-26485-4.

Disclaimer/Publisher's Note: The statements, opinions and data contained in all publications are solely those of the individual author(s) and contributor(s) and not of MDPI and/or the editor(s). MDPI and/or the editor(s) disclaim responsibility for any injury to people or property resulting from any ideas, methods, instructions or products referred to in the content.
